# Controversial COVID-19 Cures: Hydroxychloroquine and Oleander Pediatric Ingestion Simulation Cases

**DOI:** 10.7759/cureus.26176

**Published:** 2022-06-21

**Authors:** Joshua J Solano, Rebecca A Mendelsohn, Rami A Ahmed, Richard D Shih, Lisa M Clayton, Scott M Alter, Patrick G Hughes

**Affiliations:** 1 Department of Emergency Medicine, Florida Atlantic University Charles E. Schmidt College of Medicine, Boca Raton, USA; 2 Department of Emergency Medicine, Indiana University School of Medicine, Indianapolis, USA; 3 Department of Emergency Medicine, Methodist Hospital, Indianapolis, USA

**Keywords:** covid 19, simulation in medical education, pediatrics, toxicology, cardiac glycosides, oleander

## Abstract

Introduction: The use of hydroxychloroquine has dramatically increased since being touted as a potential therapeutic in combating coronavirus disease 2019 (COVID-19) caused by the SARS-CoV-2 virus. This newfound popularity increases the risk of accidental pediatric ingestion, whereby just one or two tablets causes morbidity and mortality from seizures, cardiac dysrhythmias, and cardiogenic shock. The unique management of hydroxychloroquine overdose makes it imperative for emergency medicine physicians to have familiarity with treating this condition. Similarly, ​​during the COVID-19 pandemic, there have been publicized cases touting extracts of oleander as being a potential therapeutic against the illness. Since it is commonly available and potentially lethal ingestion with a possible antidote, we developed a simulation case based on the available literature. The two cases were combined to create a pediatric toxicology curriculum for emergency medicine residents and medical students. Both of these treatments were selected as simulation cases since they were being touted by prominent national figures as potential cures for COVID-19.

Methods: Two series of simulation cases were conducted in a high-fidelity simulation lab with emergency medicine residents and medical students. The hydroxychloroquine simulation case involved the management of a four-year-old male who presented to the emergency department with nausea, vomiting, and tachycardia after ingesting hydroxychloroquine tablets. As the case unfolded, the child became increasingly unstable, eventually experiencing QT prolongation, torsades de pointes, and ventricular fibrillation arrest requiring appropriate resuscitation to achieve a return of spontaneous circulation. The oleander simulation case involved the management of a three-year-old male who presented to the emergency department with nausea, vomiting, and tachycardia after ingesting parts of an unknown plant. As that case progresses, the child becomes increasingly unstable, eventually experiencing atrial fibrillation, bradycardia, and degenerating into pulseless electrical activity and cardiac arrest requiring appropriate resuscitation to achieve the return of spontaneous circulation. Both series of simulation cases were modifiable based on trainee level and had the ability to include ancillary emergency department staff.

Results: Each simulation case was performed six times at our simulation center, with a total of 22 learners for the hydroxychloroquine case, and 14 for the oleander case. Through pre- and post-simulation confidence assessments, learners demonstrated increases in knowledge of toxidromes, evaluating pediatric overdoses, treating cardiac dysrhythmias, performing pediatric advanced life support, and managing post-arrest care. Learners also demonstrated improvements in recognizing the unique treatment of hydroxychloroquine and oleander toxicity, the toxic dose of both substances in a child, and the most common electrolyte anomaly seen in each toxicity.

Discussion: Simulation training enables learners to manage rare and complex disease processes. These cases were designed to educate trainees in recognizing and treating rare overdoses of emerging “therapeutics” that were touted early in the COVID-19 pandemic.

## Introduction

The first patients of the coronavirus disease 2019 (COVID-19) pandemic presented in the United States in early 2020. With few effective treatment options immediately available, many unproven substances began to be discussed as potential “cures” for COVID-19. Among the more promulgated theoretical therapies were hydroxychloroquine, an anti-malarial medication, and oleander, a subtropical plant, both of which were being highly touted by prominent national leaders with wide audiences over social and conventional media platforms. Concerns began to arise about potential toxic ingestions in patients taking these medications without instructions from their physicians, or the possibility of children accidentally ingesting their parents’ medications.

Hydroxychloroquine, a medication approved for use in the United States since 1955, is used to treat malaria, rheumatoid arthritis, lupus, and other conditions. While generally considered less toxic than its derivative medication, chloroquine, it has been implicated in cardiac dysrhythmias, cardiomyopathies, retinopathy, and other severe side effects in adults [1]. In a number of case reports, ingestion of hydroxychloroquine by children has caused significant morbidity and mortality from seizures, cardiac dysrhythmias, and cardiogenic shock [2]. Perhaps even more concerning, some children have experienced severe toxic effects after taking a single tablet [1,3]. After a series of toxic ingestions in children, toxicologists have agreed that a combination of diazepam, epinephrine, early intubation, and proactive supportive care can be utilized to prevent fatalities in severe hydroxychloroquine overdoses [4]. Prompt recognition of hydroxychloroquine toxicity is crucial in providing appropriate and timely care.

Prior to 2020, there was a relatively low baseline prevalence of hydroxychloroquine utilization in American households; therefore, the risk of accidental pediatric ingestion was fairly low. Considered a potential therapy against COVID-19 during the early stages of the pandemic, the magnitude of prescriptions filled nationally for hydroxychloroquine increased substantially [5]. This widespread distribution markedly increased the risk of children's potential accidental ingestion of hydroxychloroquine.

Providing instruction in both the management of hydroxychloroquine toxicity and QT-prolonging medication exposure overall, this case is ideally led by emergency medicine and pediatrics residents and fellows, while also providing useful learning for medical students. There are no known published curricula related to hydroxychloroquine toxicity. In addition, the treatment approach to hydroxychloroquine toxicity is uncommon and is best reinforced via a simulation session in which participants can learn via trial and error.

​​Unlike hydroxychloroquine, Oleander (Nerium oleander) is not a commonly prescribed medicinal, but a common plant that is found worldwide in subtropical and temperate areas. In the United States, it is found in the southern and western regions and is commonly used in residential areas as a small shrub or tree. All parts of the plant contain cardiac glycosides that have significant cardiac effects [6]. This toxicity is magnified in pediatric patients due to their smaller mass. Symptoms of severe toxicity include altered mental status, mydriasis, peripheral neuritis, qt prolongation, hypotension, heart block, atrial fibrillation, and ventricular dysrhythmias [6].

The mechanism for the toxicity stems from the cardiac glycosides that bind and inhibit the sodium/potassium ATPase pump leading to increased myocyte calcium. This leads to increased inotropy and increased extracellular potassium. Increased cardiac irritability with several different dysrhythmias has been noted [6]. Because of the cardiac impacts of oleander, several case reports of fatalities have occurred [6-8]. The use of digoxin immune fab has been shown to be helpful clinically and in the lab [7,9-10].

Oleander extract has been investigated as a remedy for cancer and as a cure for other conditions [11]. During the COVID-19 pandemic in 2020 oleander was touted as a possible treatment or preventative for COVID-19 infection by several high-profile figures in the United States [12,13]. Therefore oleander toxicity may become a concern if people attempt to utilize it as home therapy. Currently, there are no known published curricula related to oleander ingestion. The mechanism of oleander serves to discuss similar pathophysiology in digitalis overdoses. The case allows for discussion of cardiac glycosides and their mechanism and toxicity in clinical situations.

## Materials and methods

Curriculum development

Both cases were written by a panel of simulation fellowship-trained faculty, emergency medicine core faculty, and board-certified toxicologists. The facilitators consisted of the emergency medicine core faculty and members of the simulation center professional staff. The simulations occurred on separate days spaced months apart with residents and medical students. It was part of a pilot pediatric toxicology curriculum.

These cases were designed to guide learners through the management of severe, acute toxicity resulting from a hydroxychloroquine overdose and an oleander overdose, respectively. It was designed for resident and fellow physician learners who may encounter such a case in the emergency department, including pediatric emergency medicine fellows, pediatric residents, and emergency medicine residents.

Equipment/environment

The simulation cases were conducted in the simulation lab using a pediatric high-fidelity manikin. The learning management system was preloaded with the requisite vital signs, laboratory values, x-ray imaging, and electrocardiograms (EKG). Medical equipment available in the room included a crash cart, pediatric-size defibrillator pads, defibrillator, stethoscopes, medication vials, Broselow tape, intravenous (IV), interosseous (IO) supplies, endotracheal tubes, laryngoscopes, bag-valve masks, nasal- and oral-pharyngeal airways. A variety of IO and airway equipment sizes, for both pediatrics and adults, were available. Instructors verbalized all physical exam findings and patient responses to the initial history and physical exam.

Personnel

The simulation cases were ideally designed for two to five learners, with the roles somewhat flexible and able to be combined if the case is run with fewer learners. We utilized the roles of team leader, primary/secondary examiner, historian, proceduralist, and crash-cart operator. The role of the team leader is the least flexible and is ideally assumed by a physician trainee, such as a pediatric emergency medicine fellow or an emergency medicine resident. The remaining roles, including primary/secondary examiner, historian, proceduralist, crash-cart operator, etc., can be managed by physician trainees, medical students, or other health professionals. At the onset of each case, the team leader assigns each team member a role. In our simulations, we had the facilitator play the nurse role.

Assessment

Before each case began, learners were given an assessment questionnaire with five confidence-assessment questions and five knowledge-assessment questions via paper exam. These questions were developed by the faculty to emphasize key clinical concepts from the simulation scenario.

Confidence questions were utilized to track learners’ self-assessed readiness to perform skills required in the simulation, while knowledge questions determined objective knowledge acquisition and learning related to the case content. Confidence questions were rated on a five-point Likert scale, from “very unconfident” (1) to “very confident” (5). Knowledge-based questions were single-best-choice answers with 4 potential answer choices. After both the simulation and debriefing, this same assessment questionnaire was administered to learners again. Results were collected and analyzed using Excel (Microsoft) and analysis was performed by STATA (StataCorp).

A critical actions checklist was enumerated in the case, which facilitators scored as the simulation progressed. Any critical action omitted or performed erroneously by the team was discussed during the debriefing. The critical actions varied in scope, with a mixture of both broad (i.e., “Demonstrate clear communication with team members”) and narrow (“Place the patient on a cardiac monitor”) objectives.

At the end of the scenario, a debriefing session was conducted. The instructor emphasized that simulation is the place for learning, and the environment of the debrief is designed to facilitate discussion of enhancing the skills and knowledge of the entire team. After this announcement, the instructor asked the team leader to self-evaluate how the case went, and what was subjectively good and bad about the progression of the case. Later, each participant was asked to provide their insights regarding the progression of the case and the communication between the leader and the team. Following this, the instructor reviewed the critical actions list and highlighted the team’s strengths and areas for improvement. Lastly, the instructor went over the optimal management of each case, highlighting the unique therapeutics and procedures utilized in hydroxychloroquine and oleander toxicity, respectively. The entire debriefing session lasted 20 to 30 minutes for each case. The PEARLS Healthcare debriefing was the tool used as a model for the debriefing guide [14].

## Results

The hydroxychloroquine simulation case was performed at our simulation center six times, in groups of 3 to 4 learners per group with a total of 18 residents and four medical students. Each group comprised emergency medicine residents at various stages of training and medical students. All 22 learners filled out both the pre-simulation and post-simulation assessments, with one learner leaving the final three knowledge-based questions blank on both assessments.

Comparing the pre-test and post-test results using the Wilcoxon signed ranks test, there was a statistically significant increased comfort level for all differences (p<0.01 for each) (Table 1).

**Table 1:** Hydroxychloroquine confidence question results.

	Mean (SD)		Median (IQR)		P-value
	Pre	Post	Pre	Post	
Q1	2.36 (.902)	3.14 (.889)	2 (2-3)	3 (2-4)	<0.01
Q2	2.27 (1.120)	3.05 (.999)	2 (1-3)	3 (2-4)	<0.01
Q3	2.14 (.990)	3.00 (.976)	2 (1-3)	3 (2-4)	<0.01
Q4	2.18 (.958)	3.05 (.921)	2 (1-3)	3 (2.5-4)	<0.01
Q5	2.09 (.868)	2.82 (.795)	2 (1-3)	3 (2-3)	<0.01

In the hydroxychloroquine knowledge-based post-test, 100% of participants correctly identified diazepam as the antidote of choice for hydroxychloroquine toxicity; 95% correctly chose 10mg/kg as the recognized toxic dose of hydroxychloroquine in a child; 90% correctly chose 30 minutes as the time it takes for symptoms to develop after ingestion of a hydroxychloroquine overdose; 100% identified QT prolongation as the most common EKG abnormality of severe hydroxychloroquine overdose; and finally, 100% identified hypokalemia as the most common electrolyte disturbance found on initial lab work of patients experiencing hydroxychloroquine toxicity. Using the McNemar test for comparison of binomials, there was a statistically significant improvement in knowledge of the antidote to hydroxychloroquine, the toxic dose, and the most common electrolyte anomaly (p<0.01 for each), and time from ingestion to symptoms (p=0.016) (Table 2).

**Table 2:** Hydroxychloroquine knowledge question results, % correct (95% confidence intervals).

	Pre	Post	P-value
Q1	18% (5-40)	100% (85-100)	<0.01
Q2	18% (5-40)	95% (77-100)	<0.01
Q3	57% (34-78)	90% (70-99)	0.02
Q4	90% (70-99)	100% (84-100)	0.500
Q5	57% (34-78)	100% (84-100)	<0.01
Mean Score	46% (38-55)	95% (88-101)	<0.01

Of the 12 critical actions on the critical action checklist for the hydroxychloroquine case listed in Table 3, all six groups (100%) obtained IV or IO access, utilized the PALS algorithm in resuscitating the patient, admitted the patient to the intensive care unit (ICU), and demonstrated clear communication with the patient and fellow team members. Five groups (83%) recognized the patient’s decompensation to ventricular fibrillation arrest, defibrillated appropriately, and utilized appropriate weight-based dosing for medications, equipment, and interventions. Four groups (67%) performed an initial primary survey, obtained an accurate history of hydroxychloroquine ingestion, obtained an initial EKG and lab studies, placed the patient on a cardiac monitor, and contacted the poison control center for recommendations. Only three groups (50%) promptly recognized the torsades de pointes dysrhythmias and treated the torsades appropriately with magnesium sulfate.

**Table 3:** Hydroxychloroquine critical action compliance.

Action #	% (95% CI)
1 Perform initial primary survey	67% (22-96)
2 Obtain intravenous or intraosseous access	100% (54-100)
3 Obtain an accurate history to elicit hydroxychloroquine ingestion information from parents	67% (22-96)
4 Obtain an initial electrocardiogram and appropriate lab studies	67% (22-96)
5 Place patient on a cardiac monitor	67% (22-96)
6 Prompt recognition of Torsades dysrhythmia and appropriate treatment with magnesium sulfate	50% (12-88)
7 Utilize pediatric advanced life support algorithm in the resuscitation of the patient, including stabilizing airway, breathing, and circulation	100% (54-100)
8 Recognize patient’s decompensation to ventricular fibrillation arrest and defibrillate appropriately	83% (36-100)
9 Utilize appropriate pediatric weight-based dosing for medications, equipment, and interventions	83% (36-100)
10 Contact the poison control center for hydroxychloroquine-specific recommendations on epinephrine drip and high-dose diazepam	67% (22-96)
11 Admit patient to the intensive care unit	100% (54-100)
12 Demonstrate clear communication with patient’s family and with team members	100% (54-100)
Avg	79% (67-91)

The oleander simulation case was similarly performed at our simulation center six times, in groups of two to three learners per group - a total of 13 residents and 1 medical student. Each group was composed of emergency medicine residents at various stages of training and medical students. All 14 learners filled out both the pre-simulation and post-simulation assessments.

In the pre-oleander simulation assessments, the mean confidence level for evaluating accidental toxic plant ingestion in a pediatric patient and the knowledge and ability to manage a pediatric toxidrome were the lowest assessed. The antidote, timing of the toxicity, and the most common rhythm associated with toxidrome were the lowest areas for knowledge assessment. In the post-oleander toxicity simulation assessment, the mean comfort level for evaluating accidental plant ingestion in a pediatric patient was 3.5± 0.86; the mean level of confidence in managing a pediatric dysrhythmia was 3.79 ± 0.7; the mean level of confidence in managing a pediatric pulseless electrical activity was 3.93 ± 0.73; mean level of confidence stabilizing a pediatric patient after achieving the return of spontaneous circulation (ROSC) was 3.5 ± 0.76; and mean level of confidence and knowledge in their ability to manage pediatric toxidromes was 3.36 ± 0.84. Comparing the pre-test and post-test results using the Wilcoxon signed ranks test, there was a statistically significant increased comfort level for all differences (p<0.01) (Table 4).

**Table 4:** Oleander confidence questions

	Mean (SD)		Median (IQR)		P-value
	Pre	Post	Pre	Post	
Q1	2.07 (.917)	3.50 (.855)	2 (1-3)	4 (3-4)	<0.01
Q2	2.79 (.699)	3.79 (.699)	3 (2-3)	4 (3-4)	<0.01
Q3	2.86 (.949)	3.93 (.730)	3 (2-4)	4 (3-4.25)	<0.01
Q4	2.50 (.650)	3.50 (.760)	3 (2-3)	3.5 (3-4)	<0.01
Q5	1.86 (.770)	3.36 (.842)	2 (1-2.25)	3 (3-4)	<0.01

In the knowledge-based post-test of the oleander case in Table 5, 100% of participants correctly identified digoxin immune fab as the antidote of choice for oleander toxicity; 100% correctly chose digitalis as a similar overdose to oleander; 100% correctly chose 2 hours as the time it takes for symptoms to develop after ingestion of oleander; 100% identified atrial fibrillation with bradycardia as the most common EKG abnormality of severe oleander ingestion; and finally, 100% identified hyperkalemia as the most common electrolyte disturbance found on initial lab work of patients experiencing severe oleander toxicity. Using the McNemar test for comparison of binomials, there were statistically significant improvements in knowledge of all but the second question about digitalis being a similar overdose to oleander.

**Table 5:** Oleander Knowledge question results, % correct (95% Confidence Intervals)

	Pre	Post	p-value
Q1	43% (18-71)	100% (77-100)	<0.01
Q2	79% (49-95)	100% (77-100)	0.25
Q3	14% (2-43)	100% (77-100)	<0.01
Q4	14% (2-43)	100% (77-100)	<0.01
Q5	50% (23-77)	100% (77-100)	0.02
Mean score (95% CI)	40% (24-56)	100%	

Of the 12 critical actions on the critical action checklist for the oleander case in Table 6, six groups (100%) performed the initial primary assessment, obtained IV or IO access, obtained an accurate history of oleander ingestion, placed the patient on a cardiac monitor, recognized the patient’s decompensation to pulseless electrical activity, utilized appropriate weight-based dosing for medications, and admitted the patient to the ICU. Five groups (83%) contacted the poison control center for recommendations and demonstrated closed-loop communication with team members. Four groups (67%) obtained an initial EKG and lab studies.

**Table 6:** Oleander critical action compliance

Action #	% (95% CI)
1 Perform initial primary survey (including assessment of airway, breathing, circulation)	100% (54-100)
2 Obtain intravenous or intraosseous access	100% (54-100)
3 Obtain an accurate history to elicit unknown plant ingestion information from the mother	100% (54-100)
4 Obtain an initial electrocardiogram, radiological and lab studies	67% (22-96)
5 Place patient on a cardiac monitor	100% (54-100)
6 Recognition of atrial fibrillation with bigeminy and appropriate treatment with digibind	33% (4-78)
7 Utilize pediatric advanced life support bradycardia algorithm in the resuscitation of the patient, including stabilizing the airway, breathing, and circulation	33% (4-78)
8 Recognize patient’s decompensation to pulseless electrical activity if no digibind given and begin pediatric advanced life support algorithm	100% (54-100)
9 Utilize appropriate pediatric weight-based dosing for medications, equipment, and interventions	100% (54-100)
10 Contact the poison control center for unknown plant ingestion or oleander specific recommendations for digibind	83% (36-100)
11 Admit patient to the intensive care unit	100% (54-100)
12 Demonstrates closed-loop communication with team members	83% (36-100)
Avg	83% (78-89)

## Discussion

These cases were designed to teach emergency medicine residents and medical students how to resuscitate a pediatric patient after accidental ingestion of near-fatal doses of hydroxychloroquine or oleander. During the COVID-19 pandemic, hydroxychloroquine experienced a dramatic increase in availability in American households. Unfortunately, many parents and physicians were unaware of the medication’s potentially deadly risk to children who accidentally ingest even a few tablets [1]. Few physicians in the United States have previously cared for patients experiencing hydroxychloroquine toxicity, however, case reports and therapeutic guidelines are fairly well-established in the toxicology literature [4]. Early intubation, cardiovascular support, diazepam administration, epinephrine drips, and careful electrolyte monitoring, have all been described as pivotal measures for physicians to employ early in the process of resuscitating a patient with severe hydroxychloroquine toxicity [2]. While some of these measures are already part of typical resuscitation protocols, others are less commonly considered - especially the administration of diazepam, even in patients who are not actively seizing. This unique therapeutic approach made the creation and execution of this simulation case both timely and important for the learners involved.

To ensure a seamless case flow without overly cumbersome complexity, certain features of hydroxychloroquine toxicity were left out or de-emphasized but discussed during the post-simulation debriefing. For example, the case takes place more than an hour after the child ingested hydroxychloroquine. This timing was chosen to avoid the debate among toxicologists regarding the utility of gastric lavage for patients who present to the emergency department less than one hour after ingestion. Case reports on the subject demonstrate equivocal advice regarding this decision [4]. The child did not experience a seizure even though nearly half of the case reports of severe hydroxychloroquine toxicity involve seizures as a presenting symptom [2]. This decision was made primarily out of time constraints and case-flow concerns, but also to highlight the use of diazepam, even for patients who are not actively seizing. Thirdly, hydroxychloroquine commonly presents with hypokalemia [4]. However, hydroxychloroquine-induced hypokalemia is a temporary condition, transpiring while potassium is driven intracellularly [15]. Theoretically, there is a risk that once the patient stabilizes, the potassium will shift back into the serum, and severe hyperkalemia may result if the potassium has been replaced too aggressively. Case reports are mixed on the subject, as are toxicologists’ recommendations on repletion, so this aspect of the toxicity was avoided. Lastly, the case was intentionally designed to result in cardiac arrest. Even if teams performed optimal resuscitation during the early phase of the simulation, barriers were intentionally placed (such as delayed availability of medications) to ensure that the primary learning objective for the learners - assisting during a pediatric code - was a key component of the simulation.

​​Similarly, oleander ingestion was chosen as the second toxic substance because, at the time of the inception of this case during the COVID-19 pandemic, oleander and oleander extract had also been proposed as having possible curative or preventative properties to COVID-19 [12-13]. Unfortunately, many were unaware of the medication’s potentially deadly risk to children who accidentally ingest the plant, extracts, or teas [6,8]. Few physicians in the United States have previously cared for patients experiencing oleander toxicity, however, case reports and therapeutic guidelines are in the toxicology literature [6-8]. Cardiovascular support, digoxin immune fab administration, and careful electrolyte monitoring have all been described as pivotal measures for physicians to employ early in the process of resuscitating a patient with severe oleander toxicity [6,8].

In each of these two disparate simulation cases, learners demonstrated improvements in their knowledge base regarding the specific details of the respective pediatric toxidrome management. Learners also demonstrated improved confidence scores in all categories - confidence in evaluating a pediatric drug overdose, managing a pediatric cardiac dysrhythmia, managing a pediatric code, stabilizing a pediatric patient after ROSC, and managing pediatric toxidromes overall. Some learners with the lowest initial pre-simulation confidence scores reported some of the largest increases in post-simulation confidence. This may be a result of more inexperienced learners, with a lack of initial exposure to pediatric resuscitations overall, or certainly lack of exposure to these rare toxidromes, suddenly receiving a surge of information and confidence on these relevant topics. On knowledge and confidence assessments some statistically significant increases were seen, most learners ended up with an average of “3,” which equates to neutral on the scale. Since we attempted to balance teams by training level it is unclear why some performed more critical actions than others. We were unable to discern a pattern in why some groups performed better than others in the simulation.

Limitations

Perhaps ironically, the inspiration for these cases, the COVID-19 pandemic, was the biggest barrier to the implementation of the simulation. The pediatric emergency department made the decision to postpone in-situ simulations because of concern for an excess number of people congregating at the same time in a confined area. As a result, the cases were executed in a simulation lab with emergency medicine residents and medical students.

In addition, as described in the discussion section, we did not study long-term knowledge retention as part of our study. It is possible that, while short-term knowledge gains were identified, over time, that knowledge base will wane. Ideally, even if learners do not recall specifics of the case management, they will retain basic tenets, such as obtaining as much collateral information from patients and their families as possible, calling poison control for help in managing potential intoxications, and being careful when determining age-appropriate dosages and equipment sizes during pediatric resuscitations.

Lastly, both cases were intentionally designed to result in pulseless arrest. Even if teams performed optimal resuscitation during the early phases of the simulations, barriers may be intentionally placed (such as delayed availability of medications) to ensure that one of the primary learning objectives, managing a pediatric code, was a key component of the simulations.

## Conclusions

This simulation case series was developed to educate emergency physicians about the management of overdoses from popularized COVID-19 therapies. The oleander and hydroxychloroquine pediatric toxicity cases are easily performed using commonly available simulation materials. Simulation is the ideal methodology for increasing learner knowledge, skills, and attitudes about low-frequency high-risk cases such as pediatric overdoses.

## Appendices

**Table 7:** Pediatric hydroxychloroquine ingestion simulation case

PATIENT NAME: Alex PATIENT AGE: 4 years old PATIENT WEIGHT: 15 kg CHIEF COMPLAINT: “Nausea & Vomiting”	
Brief narrative description of the case	Alex is a 4-year-old male with no past medical history who complained to his parents that he was feeling “yucky” before vomiting. When his mother went to the bathroom to grab a thermometer, she noticed her hydroxychloroquine tablets were spilled out on the counter, prompting her to bring Alex straight to the Emergency Department. (ED) Upon initial evaluation in the ED, Alex is mildly tachycardic, but their vitals are otherwise stable. Initial lab values are normal, while the EKG demonstrates QT prolongation. Shortly thereafter, Alex becomes unresponsive and goes into a Torsades dysrhythmia. Anticipated interventions include primary and secondary surveys, establishing IV access, placing the patient on a cardiac monitor, recognizing the changes in the patient condition, including the dysrhythmia and eventual ventricular fibrillation arrest, and treating per Pediatric advanced life support (PALS) algorithms, including securing his airway and evaluating his breathing and circulation, defibrillation, administering appropriate medications, stabilizing the patient hemodynamically, obtaining appropriate laboratory values and electrocardiogram (EKG), and calling various consultants.
Primary Learning Objectives	By the end of this module, the learner will be able to: Demonstrate a systematic approach to the evaluation and management of a pediatric toxic ingestion Describe the signs and symptoms of hydroxychloroquine intoxication in a pediatric patient Demonstrate competence in pediatric resuscitation protocols
Critical Actions	Perform initial primary survey Obtain intravenous or intraosseous (IV/IO) access Obtain an accurate history to elicit hydroxychloroquine ingestion information from parents Obtain an initial EKG and appropriate lab studies Place patient on a cardiac monitor Prompt recognition of Torsades dysrhythmia and appropriate treatment with magnesium sulfate Utilize PALS algorithm in the resuscitation of the patient, including stabilizing airway, breathing, and circulation Recognize patient’s decompensation to ventricular fibrillation arrest and defibrillate appropriately Utilize appropriate pediatric weight-based dosing for medications, equipment, and interventions Contact the poison control center for hydroxychloroquine-specific recommendations on epinephrine drip and high-dose diazepam Admit patient to ICU Demonstrate clear communication with the patient’s family and with team members
Learner Preparation	General knowledge of toxidromes and pediatric emergency medicine PALS course competency

Initial Presentation			
Initial vital signs	Heart rate (HR) 140 bpm Oxygen saturation (SpO2) 99% Blood Pressure (BP) 95/65 mmHg Respiratory Rate (RR) 25 Temperature (T) 37.0 degrees Celsius		
Overall Appearance	Alex walks into the emergency department with his mom, carrying an emesis basin. He is wearing a t-shirt and shorts. He is led to a standard pediatric emergency department bed, where he lays down looking generally uncomfortable. He is not on any monitors when you walk in the room.		
Actors and roles in the room at case start	This scenario requires a minimum of 2 embedded participants, one to play the nurse role, and one to play the parent role. The case instructor can play the parent role if needed. 2 to 5 participants can be utilized to play the provider roles. Participant #1: Team Leader (physician) Participant #2: Airway and Procedures Lead (Optional) Participant #3: Physical Exam Lead (Optional) Participant #4: History Lead (Optional) Participant #5: Medication Prep and Administration Lead Instructor #1: Simulation facilitator who will also act as debriefer and can act in the role of parent, if the personnel is limited Instructor #2: Simulation team member who will act in the role of the nurse and can provide the team with lab values and imaging at the appropriate time and describe what equipment and medications are available		
History of Present Illness	“Alex has been vomiting non-stop for the past hour! He says he feels ‘yucky’ and has been holding his stomach. He was totally fine and playing normally just a few hours ago!” When asked about events leading up to the event (SAMPLE): SAMPLE history: Signs/symptoms (sx)- “Alex was feeling totally fine just two hours ago! But about an hour ago, he came to us and said he was feeling sick. He just didn’t look right, and then threw up on the kitchen floor. He’s now saying his belly hurts and threw up three times on the car ride here. He has been eating and drinking normally, and peeing and pooping his usual amount. Aside from nausea, vomiting, belly pain, and general ‘yucky’ feeling, he hasn’t complained about anything else.” Allergies- none Medications- none Past Medical History: “He was born full-term, no complications. His immunizations are up to date. He has never been hospitalized or had surgery. He has not had any sick contacts.” Last meal: “He had macaroni at noon.” Events preceding: “He was running around the house all day, asking me for candy, and seemed totally normal. After he vomited, I went to the bathroom to get a thermometer, and that’s when I found my pills spilled out onto the counter. I don’t know if he ate any, thinking they were candy, but that’s why I brought him here right away.” If asked for review of systems: Positive for nausea, vomiting, and abdominal pain. The parent denies fevers, headaches, eye redness or discharge, congestion, shortness of breath, chest pain, diarrhea, bloody stool, abnormal bleeding, bruising, musculoskeletal or skin abnormality. If asked about home environment/social history: He lives at home with his parents. He is in pre-kindergarten but is currently home for a school break. His parents are both currently working from home, and deny any drugs or alcohol being kept in the home. If asked about family history: Alex’s mom has a history of Lupus, for which she takes hydroxychloroquine 400mg/day. His dad has no medical problems.		
Past Medical/Surgical History	Medications	Allergies	Family History
None	None	None	Mom has Lupus and takes hydroxychloroquine 400mg/day
Initial Physical Examination			
General	Laying on the stretcher curled up, holding his stomach		
Head, Eyes, Ears, Nose, Throat	Head is normocephalic, atraumatic. Pupils are equal, round, and reactive to light. Oropharynx is normal. No lymphadenopathy.		
Neck	Supple		
Lungs	Breathing comfortably with lungs clear to auscultation bilaterally with no wheezing, rhonchi or rales		
Cardiovascular	Tachycardic with bounding peripheral pulses. 2+ capillary refill		
Abdomen	Generalized tenderness of the abdomen, but on palpation, abdomen is soft with no rebound, rigidity or guarding. No palpable masses.		
Neurological	Pupils are equal, round, and reactive to light. He is able to speak to his mom in full and complete sentences. Normal and symmetric reflexes. Patient is able to ambulate		
Skin	No rashes or lesions		
Genitourinary	Normal exam		
Psychiatric	Cooperative		

Instructor Notes - Changes and CASE Branch Points		
Intervention / Time point	Change in Case	Additional Information
Learners establish team roles *this may be done prior to entering the simulation room or immediately after entering the room.		Essential team roles: Team Lead Airway & Procedure Lead
Patient is walked over to an ED bed, where he lays down, with parent(s) at bedside.	Heart rate (HR) 140 Oxygen saturation (SpO2) 99% Blood Pressure (BP) 95/65 mmHg Respiratory Rate (RR) 25 Temperature (T) 37.0 degrees Celsius Initial visual impression: Patient dressed in shorts and a t-shirt. He is curled up next to an emesis basin, holding his stomach. He appears generally uncomfortable, but is breathing easily and is interactive with his parents and with the care team.	
History is obtained from the parent		Parent responds to questions appropriately. Initially only gives history of child vomiting and complaining of belly pain. When questioned further about family history, environment, or preceding history, describes finding hydroxychloroquine pills in the bathroom.
Assess airway, breathing, circulation (ABCs). Monitors are applied to patient, including cardiac monitor and pulse ox.	In conjunction with vital signs above: Airway: intact with patient speaking appropriately Breathing: Lungs are CTAB with normal WOB Circulation: Tachycardic with bounding pulses May apply oxygen if deemed necessary. Continuous ECG monitoring: tachycardic with prolonged QT interval	Oxygen saturation (SpO2) remains at 99% if supplemental oxygen is applied.
Assess circulation with pulse and perfusion check.	2+ distal pulses, <2 second capillary refill	
Complete primary survey including neurologic assessment and exposure.	Undress patient and perform secondary survey, which is normal aside from generalized abdominal tenderness	
IV access is obtained Participant requests labs: finger-stick glucose, electrolytes, blood urea nitrogen, creatinine, liver function tests, Tylenol, salicylate, EtOH levels, urine analysis and urine drug screen Give 20cc/kg intravenous fluid (IVF) bolus. EKG ordered		EKG and Lab results announced 3 minutes later: Point of care glucose: 111 mg/dL. Normal serum electrolytes with a sodium of 138 mEq/L, potassium of 3.6 mEq/L, chloride of 100 mEq/L, CO2 of 22, BUN 20, and creatinine of 0.9. alanine aminotransferase, *aspartate aminotransferase* , and alkaklaine phosphate were within normal limits. Tylenol level was <10 mcg/mL and salicylate level was <1 mg/dL. Blood alcohol level is zero. Urine analysis is normal, yellow in color, pH 6.0 with no RBCs, WBCs, Nitrites or Leukocytes identified. Urine drug screen is negative.
Labs have been drawn, fluid bolus begun when RN obtains EKG	As RN hands EKG to Team Leader, patient complains he’s feeling sick again and begins to vomit	EKG demonstrates QT prolongation with a QTc of 500ms
If the team does not recognize QT prolongation, they may consider an anti-emetic such as Ondansetron.	If team gives Ondansetron at any point, cardiac monitor immediately changes to a torsades morphology	
CXR, abdominal flat and decubitus Xray requested Abdominal computed tomography (CT) requested Abdominal ultrasound requested Poison Control Center called Diazepam may be ordered		“Xray is en route.” “There is another patient in head CT right now, it will be at least 5 minutes until they are available.” “Ultrasound won’t be available for at least an hour.” “You are currently on hold with the poison control center” “The nurse is calling the pharmacy now to try to get the diazepam”
3-5 MINUTES INTO THE CASE		
Reassess airway, breathing, circulation (ABCs).	Patient is unresponsive. He is breathing agonally with a thready pulse. Torsades morphology is apparent on the cardiac monitor.	Parent asks “What just happened? What’s going on with my son?!” If multiple team members, one member pulls parent aside and calmly explains everything that is happening regarding interventions and patient’s evolving status.
The team orders magnesium sulfate and addresses Airway, Breathing and Circulation again. BVM is used to oxygenate patient while advanced airway equipment is gathered at bedside. Code cart is brought to the bedside, and pediatric pads are applied to patient’s chest.	As the team begins to address the Torsades, the patient no longer has palpable pulses and the rhythm on the monitor deteriorates to a ventricular fibrillation arrest	If the team fails to detect the ventricular fibrillation cardiac arrest, the embedded participant nurse can call out, “I can’t feel a pulse!” to alert the team to the change in patient status
Using the PALS algorithm for ventricular fibrillation arrest, the team immediately orders defibrillation at 2 joules/kg and then begins chest compressions. They continue to follow the PALS algorithm, including pulse checks every 2 minutes, and shock again after first pulse check, at 4 joules/kg, and give 0.01mg/kg IV of Epinephrine every 3-5 min. By third pulse check, the team considers giving Amiodarone 5mg/kg IV	The patient is pulseless and in ventricular fibrillation arrest through the first three pulse checks. If PALS is followed appropriately, after epinephrine and amiodarone are given, at pulse check #4, return of spontaneous circulation (ROSC) is established. If PALS is not followed appropriately, (eg. if the team does not shock the patient’s ventricular fibrillation rhythm, or does not give appropriate medications) the patient’s rhythm deteriorates to asystole and the case ends.	
If not already done so, the patient’s airway is definitively secured with intubation. Chest x-ray is obtained to verify placement of ET tube, and patient is connected to a ventilator. The patient’s vitals are re-assessed. Hypotension is addressed by starting an epinephrine drip at 0.01 – 1mcg/kg/min. Sedating and analgesia medications are ordered. ABG and repeat labs are ordered. Repeat EKG is ordered. Appropriate consults, including ICU, are called. Team connects with poison control center	Patient is back to a perfusing rhythm and has weak pulses. Vital signs are now: HR: 70, RR: 15 (BVM or ventilator), BP: 75/45, Temp: 37.0 degrees celsius	Intubation attempt is successful. Chest x-ray demonstrates tube in appropriate position. After epinephrine drip is initiated, the patient’s blood pressure rises to 90/50. ABG demonstrates a pH: 7.10, PaCO2: 60, PaO2: 80. All other labs consistent with prior labs. Repeat EKG demonstrates sinus rhythm. Poison Control Center answers the phone, “Hi! This is the State Poison Control Center toxicologist. How can I help today?” A team member summarizes the case for the consultant. Consultant may discuss possibility of activated charcoal with the team, but will decide against it once timeline of ingestion >1 hour ago is established. The consultant will also discuss beginning an epinephrine drip if not already performed, and will discuss administering diazepam 0.15mg/kg, even in the absence of seizures, for the theoretical cardioprotective effects in hydroxychloroquine overdose. Pediatric Intensivist returns call, “Hi! This is the pediatric intensivist. How can I help?” A team member summarizes the case. Consultant thanks the team, accepts the patient for admission, and the case ends.
Ideal Scenario Flow Ideally, the learners assign team roles outside the room and observe the patient walking in to the room. When the learners enter, they promptly obtain the patient’s vital signs, which demonstrate tachycardia, but are otherwise normal. Team members obtain a history and physical exam while also initiating obtaining IV access and placing patient on cardiac, blood pressure, and pulse ox monitoring. Ideally, the team elicits the information about the found hydroxychloroquine tablets from the patient’s mother. After IV access is secured, initial labs are obtained and IV fluids are ordered. Initial labs are within normal range for the patient’s age. EKG is ordered and demonstrates QTc prolongation to 500ms. Ideally, the team recognizes the need to obtain an EKG, blood glucose, serum electrolytes, liver function tests, acetaminophen, salicylate, EtOH levels and a UDS to assess for the broad range of sequelae from a hydroxychloroquine overdose and to assess for other medications that may have been consumed in addition to the hydroxychloroquine. The team may wish to obtain additional imaging to rule out alternate causes of abdominal pain and vomiting, but will be re-directed by the embedded participant nurse. The team may also consider administering activated charcoal, but as the ingestion has occurred longer than 1 hour prior to presentation, the intervention will provide minimal effect, and the case will continue as planned. When the patient begins to vomit, the team may choose to intervene by administering anti-emetics. If the prolonged QTc is overlooked and a medication such as ondansetron is ordered, the patient’s rhythm will immediately become torsades de pointes. The patient must be reassessed numerous times and the team will discover that the patient’s clinical status has deteriorated to a torsades dysrhythmia with faint pulses and agonal breathing. The team will work to calmly keep the family informed of the patient’s status while simultaneously working to address the airway, breathing, and circulation and resuscitate the patient appropriately. Ideally, a bag-valve mask will be utilized until a more advanced airway can be secured. The team will administer magnesium sulfate in an attempt to stop the torsades rhythm. The team will also anticipate the next steps, and will bring advanced airway equipment and the crash cart to the bedside, and will place pediatric pads on the patient. Despite the team’s best efforts, the patient will decompensate further to a ventricular fibrillation arrest. Upon loss of pulses, the team will recognize further decompensation in the patient’s status, and will correctly identify ventricular fibrillation as a shockable rhythm. Following the PALS algorithm, the team will defibrillate the patient with weight-corrected joules and will subsequently begin compressions. They will conduct pulse checks every 2 minutes, administer weight-adjusted epinephrine every 3-5 minutes, and will administer at least one weight-adjusted dose of amiodarone. By the fourth pulse check, if the PALS algorithm is followed appropriately, ROSC will be established. Post-ROSC care will be initiated. If not already performed, the patient will be successfully intubated and connected to a ventilator. A chest x-ray will confirm proper tube placement. The patient’s vitals will be reassessed, which will reveal that the patient is hypotensive. The team will ideally correct with an epinephrine drip. Subsequent labs, including an ABG demonstrate acidosis and continued hypokalemia. All other labs and imaging are within normal limits. Repeat EKG is now sinus rhythm. The local poison control center and other consultants, including the pediatric ICU team, are consulted. The poison control center advises against using activated charcoal, but does encourage the administration of an epinephrine drip and of high-dose diazepam for cardio-protective benefits in hydroxychloroquine overdose. A summary of the case is provided to the PICU consultants, the patient is admitted to the PICU, and the case ends.		Anticipated Management Mistakes Failure to elicit the hydroxychloroquine history from the parents Failure to consider additional medication ingestions and alternate diagnoses Failure to obtain an EKG to discover QTc prolongation Administration of QTc prolonging medications despite evidence of extant QTc prolongation Failure to recognize Torsades de Pointes as the patient’s initial dysrhythmia Failure to recognize that the patient no longer has palpable pulses or identify the initial arrest rhythm as a shockable ventricular fibrillation arrest Failure to dose medications and interventions for a pediatric patient Failure to contact the Poison Control Center, or to give diazepam

**Table 8:** Pediatric toxicology hydroxychloroquine overdose simulation ROSC: Return of spontaneous circulation

Pre-Simulation	Post-Simulation

	Very Unconfident	Unconfident	Neutral	Confident	Very Confident
1. How confident do you feel in your ability to evaluate an accidental medication overdose in a pediatric patient?					
2. How confident are you in your ability to manage a pediatric cardiac dysrhythmia?					
3. How confident are you in your ability to manage a pediatric code?					
4. How confident are you in your ability to stabilize a pediatric patient after achieving ROSC?					
5. How confident are you in your knowledge and ability to manage pediatric toxidromes?					

**Table 9:** Pediatric oleander ingestion simulation case

PATIENT NAME: Caleb PATIENT AGE: 3 years old PATIENT WEIGHT: 13 kg CHIEF COMPLAINT: “Nausea, Vomiting and Diarrhea”	
Brief narrative description of case	Caleb is a 3-year-old male with history of autism spectrum disorder who reports nausea, vomiting, and diarrhea. He also reports a funny feeling in his chest and a change in his vision. He was unsupervised in the backyard and may have ingested some seeds from their bushes. His mother reported he did not have any symptoms until about an hour ago. Upon initial evaluation Caleb is tachycardic and normotensive. Initial labs show hyperkalemia and the initial EKG shows atrial fibrillation with ventricular bigeminy. The case progresses to atrial fibrillation with a slowed ventricular response with bradycardia. The case will require primary and secondary surveys, establishing intravenous (IV) access, continuous cardiopulmonary monitoring, and recognition and management of the toxidrome of oleander. Critical actions will include securing an airway. Treatment PALS algorithm for pediatric bradycardia and then pediatric asystole. Anticipated interventions include primary and secondary surveys, establishing IV access, placing patient on a cardiac monitor, recognizing the changes in patient condition, including the dysrhythmia and eventual pulseless electrical activity arrest if treatment with digoxin immune fab is delayed. Treatment will include securing his airway and evaluating his breathing and circulation, administering appropriate medications including digoxin immune fab. There will be an expectation to obtain appropriate laboratory values and EKG, and calling various consultants including poison center and intensive care unit.
Primary learning objectives	By the end of this module, the learner will be able to: Describe the signs, symptoms, and treatment of oleander intoxication in a pediatric patient Demonstrate a systematic approach to the evaluation and management of pediatric toxic ingestion Demonstrate competence in pediatric bradycardia pulseless electrical activity and/or asystole management
Critical actions	Perform initial primary survey (including ABCDE, GCS) Obtain IV or intraosseous (IO) access Obtain an accurate history to elicit unknown plant ingestion information from mother, then obtain plant type from father Obtain an initial EKG and appropriate lab studies Place patient on a cardiac monitor Prompt recognition of digitalis-like effect and dysrhythmia of atrial fibrillation with bigeminy and appropriate treatment with digoxin immune fab if recognized Utilize pediatric advanced life support (PALS) algorithm in resuscitation of patient, including stabilizing airway, breathing and circulation Recognize patient’s decompensation to pulseless electrical activity/ asystole arrest and treat appropriately while searching for reversible cause Utilize appropriate pediatric weight-based dosing for medications, equipment, and interventions Contact the poison control center for oleander-specific recommendations including digoxin immune fab dosing Admit patient to ICU Demonstrate closed loop communication with patient’s family and with team members
Learner preparation	General knowledge of toxidromes and pediatric emergency medicine PALS course competency

Initial Presentation			
Initial vital signs	Heart rate (HR) 95 bpm Oxygen saturation (SpO2) 99% Blood Pressure (BP) 95/65 mmHg Respiratory Rate (RR) 25 Temperature (T) 37.4 degrees Celsius		
Overall appearance	Caleb walks into the emergency department with his mom, carrying an emesis basin. He is wearing a t-shirt and shorts. He is led to a standard pediatric emergency department bed, where he lays down looking generally uncomfortable. He is not on any monitors when you walk in the room.		
Actors and roles in the room at case start	This scenario requires a minimum of 2 embedded participants, one to play the nurse role, and one to play the parent role. The case instructor can play the parent role if needed. 2 to 5 participants can be utilized to play the provider roles. Participant #1: Team Leader (physician) Participant #2: Airway and Procedures Lead (Optional) Participant #3: Physical Exam Lead (Optional) Participant #4: History Lead (Optional) Participant #5: Medication Prep and Administration Lead Instructor #1: Simulation facilitator who will also act as debriefer and can act in the role of parent, if personnel is limited Instructor #2: Simulation team member who will act in the role of nurse and can provide the team with lab values and imaging at the appropriate time and describe what equipment and medications are available		
History of Present Illness	“Caleb has been vomiting and having diarrhea for a few hours. He had been playing outside in our yard. He may have eaten some seeds from the bushes we have.” When asked about events leading up to the event (SAMPLE): SAMPLE history: Signs/symptoms (sx)- “Caleb was acting normally 3 hours ago. He was outside playing and then came into the house. He started vomiting and then developed diarrhea. He also said he could not see right and that made me concerned. He has never complained of anything like that before. He does have a habit of putting everything in his mouth.” Allergies- none Medications- none Past Medical History: “He has autism. He was born full term, no complications. His immunizations are up to date. He was hospitalized for endoscopy after swallowing a nickel. He has never had surgery. He has not had any sick contacts.” Last meal: “He had grilled cheese at noon.” Events preceding: “We have new plants that are producing seeds and he tried to put some in his mouth the other day, but I stopped him. He is very curious and is a gustatory learner. Our backyard is well fenced so I let him play out there after lunch unsupervised.” If asked for review of systems: Positive for nausea, vomiting, abdominal pain, diarrhea and blurry vision. Parent denies fevers, headaches, eye redness or discharge, congestion, shortness of breath, chest pain, bloody stool, abnormal bleeding, bruising, musculoskeletal or skin abnormality. If asked about home environment/social history: He lives at home with his parents. He often plays in his fenced in yard. His mother is currently working from home, father is on a business trip and deny any drugs in the house, Alcohol is kept in the home on shelves. If asked about plants: Will require call to husband who will say the new bushes are oleander. If asked about family history: Parents have no significant family history		
Past medical/surgical history	Medications	Allergies	Family history
None	None	None	None
Initial physical examination			
General	Laying on the stretcher, in no distress		
Head, Eyes, Ears, Nose, Throat	Head is normocephalic, atraumatic. Pupils are equal, round, and reactive to light. Oropharynx is normal. No lymphadenopathy.		
Neck	Supple		
Lungs	Breathing comfortably with lungs clear to auscultation bilaterally with no wheezing, rhonchi or rales		
Cardiovascular	Normal rate, irregular 2+ capillary refill		
Abdomen	Epigastric tenderness of the abdomen, but on palpation, abdomen is soft with no rebound, rigidity or guarding. No palpable masses.		
Neurological	Pupils are equal, round, and reactive to light. He is able to speak to his mom in full and complete sentences. Normal and symmetric reflexes. Patient is able to ambulate		
Skin	No rashes or lesions		
Gentiourinary	Normal exam		
Psychiatric	Cooperative		

Instructor Notes - Changes and CASE Branch Points		
Intervention / Time point	Change in case	Additional information
Learners establish team roles *this may be done prior to entering the simulation room or immediately after entering the room.		Essential team roles: Team Lead Airway & Procedure Lead
Patient is walked over to an ED bed, where he lays down, with parent at bedside.	Heart rate (HR) 95 Oxygen saturation (SpO2) 99% Blood Pressure (BP) 95/65 mmHg Respiratory Rate (RR) 25 Temperature (T) 37.4 degrees Celsius Initial visual impression: Patient dressed in shorts and t-shirt. He is curled up next to an emesis basin, holding his stomach. He appears generally uncomfortable, but is breathing easily and is interactive with his parents and with the care team.	
History is obtained from the parent		Parent responds to questions appropriately. Initially only gives history of child vomiting, diarrhea, visual changes, and complaining of belly pain. When questioned further about family history, environment, or preceding history, describes the bushes and possible plant ingestion.
Assess airway, breathing, circulation (ABCs). Monitors are applied to patient, including cardiac monitor and pulse ox.	In conjunction with vital signs above: Airway: intact with patient speaking appropriately Breathing: Lungs are clear to ascultaiotion bilaterally with normal WOB Circulation: Normal rate, irregular rhythm with bounding pulses May apply oxygen if deemed necessary. Continuous ECG monitoring: atrial fibrillation with bigeminy	Oxygen saturation (SpO2) remains at 99% if supplemental oxygen is applied.
Assess circulation with pulse and perfusion check.	2+ distal pulses, <2 second capillary refill	
Complete primary survey including neurologic assessment and exposure.	Undress patient and perform secondary survey, which is normal aside from epigastric abdominal tenderness	
IV access is obtained		
Participant requests labs: finger-stick glucose, electrolytes, blood urea nitrogen, creatinine, liver function tests, Tylenol, salicylate, EtOH levels, urine analysis and urine drug screen		Point of care glucose: 95 mg/dL. Other lab results announced 6 minutes later:
Give 20cc/kg intravenous fluid (IVF) bolus.		
EKG ordered		EKG results announced 3 minutes later: ekg#1
Labs have been drawn, fluid bolus begun when RN obtains EKG	As RN hands EKG to Team Leader, patient complains he’s feeling sick again and begins to vomit	EKG demonstrates atrial fibrillation with predominant ventricular bigeminy
CXR, Abdominal computed tomography (CT) requested Abdominal ultrasound requested Poison Control Center called digoxin immune fab may be ordered		“Xray is en route.” “Multiple traumas in CT right now, it will be at least 30 minutes until they are available.” “Ultrasound won’t be available for at least an hour.” “You are currently on hold with the poison control center” “The nurse is calling the pharmacy now to try to get the digoxin immune fab”
4-6 MINUTES INTO THE CASE		
Labs result at 6 minutes		Normal serum electrolytes with a sodium of 145 mEq/L, potassium of 5.8 mEq/L, chloride of 100 mEq/L, CO2 of 25, BUN 15, and Creatinine of 0.8. ALT, AST, Alk Phos were within normal limits. Tylenol level was <10 mcg/mL and salicylate level was <1 mg/dL. Dig level if ordered is “elevated” requiring another sample. Blood alcohol level is zero. Urine analysis is normal, yellow in color, pH 6.0 with no RBCs, WBCs, Nitrites or Leukocytes identified. Urine drug screen is negative.
Reassess airway, breathing, circulation (ABCs) patient enters symptomatic bradycardia	Patient is unresponsive. Patient has agonal respirations with a thready pulse. Atrial fibrillation with bradycardia and PVCs is apparent on the cardiac monitor. Report new ekg is ekg#2	Parent asks “Caleb? He isn’t responding! If multiple team members, one member pulls parent aside and calmly explains everything that is happening regarding interventions and patient’s evolving status.
Treatment of Hyperkalemia with insulin/dextrose, calcium chloride or calcium gluconate and/or albuterol should be initiated		No change with these interventions
The team orders digoxin immune fab, epinephrine, or atropine and addresses Airway, Breathing and Circulation again. BVM is used to oxygenate patient while advanced airway equipment is gathered at bedside. Code cart is brought to the bedside, and pediatric pads are applied to patient’s chest.	As the team begins to address the bradycardia, the patient no longer has palpable pulses and the rhythm on the monitor deteriorates to a pulseless electrical activity (PEA) unless digoxin immune fab given. If epinephrine or atropine used patient course will go to PEA.	If the team fails to detect the PEA, the embedded participant nurse can call out, “I can’t feel a pulse!” to alert the team to the change in patient status
Using the PALS algorithm for PEA, begins chest compressions. They continue to follow the PALS algorithm, including pulse checks every 2 minutes, and give 0.01mg/kg IV of Epinephrine every 3-5 min. *Parents may prompt is this all from eating the plant?	The patient is pulseless and in PEA through the first two pulse checks. If PALS is followed appropriately, after epinephrine and digoxin immune fab are given, at pulse check #3, ROSC is established. If PALS is not followed appropriately, (eg. if the team does attempts to shock the patient’s PEA, or does not give appropriate medications) the patient’s rhythm deteriorates to asystole and the case ends.	
If Poison center has not been consulted, consider prompting, could this be due to a toxic plant?		Poison Control Center answers the phone, “Hi! This is the State Poison Control Center toxicologist. How can I help today?” A team member summarizes the case for the consultant. Consultant should discuss use of digoxin immune fab and may discuss possibility of activated charcoal with the team, but will decide against it once timeline of ingestion >1 hour ago is established. The consultant will also discuss beginning an epinephrine drip if not already performed
If not already done so, the patient’s airway is definitively secured with intubation.		Tube confirmation with end tidal CO2 and bilateral breath sounds
Chest x-ray is obtained to verify placement of ET tube, and patient is connected to a ventilator. The patient’s vitals are re-assessed. Hypotension is addressed by starting an epinephrine drip at 0.01 – 1mcg/kg/min. Sedating and analgesia medications are ordered. ABG and repeat labs are ordered. Repeat EKG is ordered. Appropriate consults, including ICU, are called. Team connects with poison control center	Patient is back to a perfusing rhythm and has weak pulses. Vital signs are now: HR: 60, RR: 15 (BVM or ventilator), BP: 75/45, Temp: 37.8 degrees Celsius	After epinephrine drip is initiated, the patient’s blood pressure rises to 90/50. ABG demonstrates a pH: 7.20, PaCO2: 60, PaO2: 500. All other labs consistent with prior labs. Repeat EKG demonstrates sinus rhythm. Pediatric Intensivist returns call, “Hi! This is the pediatric intensivist. How can I help?” A team member summarizes the case. Consultant thanks the team, accepts the patient for admission, and the case ends.
Ideal scenario flow Ideally, the learners assign team roles outside the room and observe the patient walking into the room. When the learners enter, they should promptly obtain the patient’s vital signs, which demonstrate tachycardia, but are otherwise normal. Team members should obtain a history and physical exam while also obtaining IV access and placing patient on cardiac, blood pressure, and pulse ox monitoring. Ideally, the team elicits the information about the plant ingestion from the patient’s mother. After IV access is secured, initial labs are obtained and IV fluids are ordered. Initial labs are within normal range for the patient’s age. EKG is ordered and demonstrates atrial fibrillation with bigeminy. The team should obtain an EKG, blood glucose, serum electrolytes, liver function tests, acetaminophen, salicylate, EtOH level, digoxin level, and a UDS to assess for the broad range of sequelae from a unknown plant ingestion. The team may wish to obtain additional imaging to rule out alternate causes of abdominal pain and vomiting, but should be re-directed by the embedded participant nurse. The team may also consider administering activated charcoal, but as the ingestion has occurred longer than 1 hour prior to presentation, the intervention will provide minimal effect, and the case will continue as planned. When the patient begins to vomit, the team may choose to intervene by administering anti-emetics. The patient must be reassessed numerous times and the team will discover that the patient’s clinical status has deteriorated to a atrial fibrillation with slow ventricular response with faint pulses and agonal breathing. The team should work calmly to keep the family informed of the patient’s status while simultaneously working to address the airway, breathing, and circulation and resuscitate the patient appropriately. The team should start compressions with a heart rate less than 60 and evidence of poor perfusion. A bag-valve mask should be utilized until a more advanced airway is secured. The team should administer digoxin immune fab to stop the atrial fibrillation with slow ventricular response. The team should anticipate the next steps. They should bring advanced airway equipment and the crash cart to the bedside, and place pediatric pads on the patient. Despite the team’s best efforts, the patient will decompensate further to a PEA arrest unless digoxin immune fab is given. Upon loss of pulses, the team should recognize further decompensation in the patient’s status and correctly identify PEA as a non-shockable rhythm. Following the PALS algorithm, the team should continue searching for the underlying cause and subsequently begin compressions. They should conduct pulse checks every 2 minutes, administer weight-adjusted epinephrine every 3-5 minutes. By the third pulse check, if the PALS algorithm is followed appropriately and digoxin immune fab is given, ROSC will be achieved. Post-ROSC care should be initiated. If not already performed, the patient should be successfully intubated and connected to a ventilator. A chest x-ray will confirm proper tube placement. The patient’s vitals should be reassessed, which will reveal that the patient is hypotensive. The team should ideally correct with an epinephrine drip. Repeat EKG is now sinus rhythm. The local poison control center and other consultants, including the pediatric ICU team should be consulted. The poison control center recommends 20 vials of digoxin immune fab. It also advises against using activated charcoal, but does encourage the administration of an epinephrine drip in the setting of oleander plant ingestion. A summary of the case is provided to the PICU consultants, the patient is admitted to the PICU, and the case ends.		Anticipated management mistakes Failure to elicit the oleander plant ingestion history from the parents: We found that this was often an omitted portion of the history and they did not attempt to utilize more resources to obtain the information. Failure to consider additional medication ingestions and alternate diagnoses: Some learners had difficulty listing multiple possible diagnosis. Failure to administer digoxin immune fab: This was a difficult cognitive leap for some groups given their rare exposure to this antidote and the lack of recognition of the cardiac glycoside toxicity. Failure to recognize change in rhythm to atrial fibrillation with slow ventricular response: This can be difficult if the monitor is not closely tracked by the trainees. Prompting can occur from the facilitator. Failure to recognize that the patient requires CPR due to bradycardia and hemodynamic compromise: This directive from PALS is often missed and trainee will wait for patient to be pulseless like ACLS. This distinction should be emphasized. Failure to dose medications and interventions for a pediatric patient: The broselow tape should be emphasized or other aides to make sure pediatric doses are used. Failure to contact the Poison Control Center: Care of the poisoned patient should involve the poison center as a routine. This point should be emphasized.

**Table 10:** Knowledge assessment (select one answer for each question)

Question	Possible Answers
1. Which medication is administered as an antidote in patients experiencing serious adverse effects of hydroxychloroquine toxicity?	a. Haloperidol b. Diazepam c. Lorazepam d. Midazolam
2. Without medical intervention, what is the commonly accepted toxic dose of hydroxychloroquine in a child?	a. 1mg/kg b. 5mg/kg c. 10mg/kg d. 50mg/kg
3. From time of ingestion, how long does it take for symptoms to appear in a severe hydroxychloroquine overdose?	a. 15 minutes b. 30 minutes c. 2 hours d. 6 hours
4. What is the most common abnormality seen on EKG with severe hydroxychloroquine overdose?	a. QT prolongation b. Supraventricular Tachycardia c. Ventricular Fibrillation d. Sinus Bradycardia
5. What is the most common electrolyte disturbance found on initial lab work in patients with hydroxychloroquine toxicity?	a. Hypocalcemia b. Hyponatremia c. Hypomagnesemia d. Hypokalemia
Answers:	1. b 2. c 3.b 4.a 5.d

**Table 11:** Knowledge assessment for oleander case (select one answer for each question)

Question	Answers
Which medication is administered as an antidote in patients experiencing serious adverse effects of oleander toxicity?	Midazolam Haloperidol Carnitine Digoxin Immune Fab (Digibind)
What unintentional overdose is oleander most likely to resemble?	topiramate valproic acid digitalis metoprolol
From time of ingestion, how long does it take for symptoms to appear in an oleander ingestion?	5 minutes 30 minutes 2 hours 72 hours
What is the most common abnormality seen on EKG with severe oleander overdose?	QT prolongation Atrial fibrillation with bradycardia Ventricular Fibrillation Sinus Bradycardia
5.What is the most common electrolyte disturbance found on initial lab work in patients with oleander toxicity?	a. Hypokalemia b. Hyponatremia c.Hypocalcemia d. Hyperkalemia
Answers:	d c c b 5. d

**Table 12:** Hydroxychloroquine critical actions checklist ABCDE: Airway, Breathing, Circulation, Disability, Exposure; GCS: Glasgow Coma Scale; IV: Intravenous; IO: interosseous; PALS: Pediatric Advanced Life Support;

Critical Actions	Performed Completely	Not Performed/Incomplete
Perform initial primary survey (including ABCDE, GCS)		
Obtain IV/IO access		
Obtain an accurate history to elicit hydroxychloroquine ingestion information from parents		
Obtain an initial EKG and appropriate lab studies		
Place patient on a cardiac monitor		
Prompt recognition of Torsades dysrhythmia and appropriate treatment with magnesium sulfate		
Utilize PALS algorithm in resuscitation of patient, including stabilizing airway, breathing and circulation		
Recognize patient’s decompensation to ventricular fibrillation arrest and defibrillate appropriately		
Utilize appropriate pediatric weight-based dosing for medications, equipment, and interventions		
Contact the poison control center for hydroxychloroquine-specific recommendations on epinephrine drip and high-dose diazepam		
Admit patient to intensive care unit		
Demonstrate clear communication with patient’s family and with team members		

**Table 13:** Pediatric toxicology oleander overdose assessment

Pre-Simulation	Post-Simulation

	Very Unconfident	Unconfident	Neutral	Confident	Very Confident
1. How confident do you feel in your ability to evaluate an accidental toxic plant ingestion in a pediatric patient?					
2. How confident are you in your ability to manage a pediatric cardiac dysrhythmia?					
3. How confident are you in your ability to manage a pediatric asystole/pulseless electrical activity algorithm of pediatric advanced cardiac life support?					
4. How confident are you in your ability to stabilize a pediatric patient after achieving return of spontaneous circulation?					
5. How confident are you in your knowledge and ability to manage pediatric toxidromes?					

**Table 14:** Oleander simulation critical actions checklist IV: Intravenous; IO: Interossessous

Critical Actions	Performed Completely	Not Performed/Incomplete
Perform initial primary survey (including assessment of airway, breathing, circulation, disability, and exposure of the patient, glucose)		
Obtain IV/IO access		
Obtain an accurate history to elicit unknown plant ingestion information from mother		
Obtain an initial EKG, radiological and lab studies		
Place patient on a cardiac monitor		
Recognition of atrial fibrillation with bigeminy and appropriate treatment with digibind		
Utilize PALS bradycardia algorithm in resuscitation of patient, including stabilizing airway, breathing and circulation		
Recognize patient’s decompensation to pulseless electrical activity if no digibind given and begin PALS algorithm		
Utilize appropriate pediatric weight-based dosing for medications, equipment, and interventions		
Contact the poison control center for unknown plant ingestion or oleander specific recommendations for digibind		
Admit patient to intensive care unit		
Demonstrates closed loop communication with team members		
